# Subjective sleep pattern in hospitalized patients

**DOI:** 10.5935/1984-0063.20220010

**Published:** 2022

**Authors:** Reijane Oliveira Lima, Maurício Batista Paes Landim, Luana Gabrielle de França Ferreira, Jivago Gentil Moreira Pinto, Nayla Raabe Venção de Moura, Marcela Flávia Lopes Barbosa

**Affiliations:** 1 Universidade Federal do Piauí, Centro de Ciências da Saúde - Teresina - Piauí - Brazil.; 2 Hospital Universitário da Universidade Federal do Piauí, Clínicas médica e cirúrgica - Teresina - Piauí - Brazil.

**Keywords:** Sleep, Sleep deprivation, Inpatients

## Abstract

**Objective:**

To evaluate the subjective sleep pattern in hospitalized patients.

**Methods:**

This is a cross-sectional design developed with 230 patients in a university hospital in northeastern Brazil from September 2017 to March 2018. We included patients 18 years old or older, hospitalized for a minimum of 48 hours and a maximum of five days, with stable clinical conditions and preserved guidance. For data collection, a structured questionnaire was used and the subjective sleep pattern was assessed using Visual Analog Sleep Scales. Univariate and bivariate statistics were calculated using IBM® SPSS® software, version 23.0.

**Results:**

In the hospitalization, the Disorder, Effectiveness, and Supplementation scales reached scores of 208.7 points, 353.8 points, and 62.9 points, respectively. The clinical characteristics that interfered with sleep were the practice of regular physical activity (p = 0.024) and a higher body mass index (p = 0.033). We also observed statistically significant differences between VAS scores and factors influencing sleep.

**Conclusion:**

There was a certain level of sleep disturbance during the hospitalization period, and consequently the need for sleep supplementation during the day. We also observed that some factors negatively influenced the quality of hospital sleep.

## INTRODUCTION

Hospitals are usually environments where getting good quality sleep is a challenge^1^. Sleep during hospitalization may not be restful or restorative and the reasons have a multifactorial etiology, including psychological stress, noise, medication, light, frequent night interventions by the multidisciplinary team, collection of laboratory tests, pain, and others^2,3^.

The stress caused by the hospitalization process brings a potentially traumatic experience, removing the individual from his daily routine and motivating a confrontation with pain and physical limitation, which can result in sleep changes during the hospitalization period^4^. Sleep disorders can affect different levels of the body. As a consequence of sleep deprivation, we highlight changes in the immune system, changes in the mechanisms of homeostasis and the neuroendocrine system, psychological damage, in addition to the increase in morbidity and mortality of patients in a hospital environment^5^.

We should pay attention to changes in the quality of sleep in hospitalized patients, as the early identification of such events may reduce numerous losses to the clinical evolution of patients during the hospitalization period. For this, the literature shows several instruments aimed at tracing the individual’s sleep pattern or obtaining information about specific sleep conditions^6^. Subjective methods to evaluate sleep, evaluate a larger number of patients and interventions, including for longer periods than those methods using polysomnography^7^.

The Visual Analog Sleep (VAS) Scales was validated in Brazil based on the original English Verran and Snyder-Halpern (VSH) sleep scale. It assesses the sleep of the last 24 hours subjectively, applying to hospitalized patients^8,9^.

Considering the sleep deprivation as a problem in the hospital environment, the evaluation of the subjective sleep pattern of hospitalized patients favors the identification of changes in the sleep-wake pattern of hospitalized patients. Therefore, this study aims to evaluate the subjective sleep pattern of patients hospitalized in a university hospital.

## MATERIAL AND METHODS

This is a cross-sectional and analytical study developed in the wards of the medical and surgical clinics of the University Hospital of the Federal University of Piauí located in the city of Teresina (PI), from September 2017 to March 2018.

Participants should be 18 years old or older; hospitalized for at least 48 hours and at most five days; with stable clinical conditions, a preserved orientation, and the ability to verbalize. The exclusion criteria were recent postoperative (up to 24 hours); hospitalization in the last 30 days; the use of a prescription or self-medication to treat sleep disorders; as well as having any disease that affected sleep such as insomnia, apnea, depression; as well as having any disease that affected sleep such as insomnia, apnea, depression; with hearing or visual impairment; and being a neurological (clinical and surgical) and psychiatric patient. The sampling was non-probabilistic for convenience, in which we included all patients who attended the service and met the inclusion criteria.

We used two instruments to collect the data: structured questionnaire; and the Visual Analog Sleep (VAS) Scales. The structured questionnaire had questions about age, gender; personal history (smoking, drinking, physical activity); medical record data such as the cause and length of stay, anthropometric measures (weight, height, body mass index - BMI). Also, patients were asked about the discomfort caused by factors influencing sleep, such as noise, lighting, temperature, bed comfort, organic disorders (pain, diarrhea, nausea, among others), care received.

For the analysis of the body mass index (BMI), the classification adapted by the World Health Organization was used, including the following cutoff points: thin or underweight (BMI <18.5 kg/m^2^), eutrophic (BMI of 18, 5 to 24.9 kg/m^2^), overweight or pre-obese (BMI 25.0 to 29.9 kg/m^2^) and obesity (BMI≥30 kg/m^2^)^10^.

The sleep analysis in the hospital was performed using the Brazilian version of the VAS Scales which obtained satisfactory internal consistency values for the Disturbance (α=0.80), Effectiveness (α=0.78) and Supplementation (α=0.72) scales, and assesses the subjective sleep of individuals hospitalized in the last 24 hours^9^ developed to assess the subjective sleep of individuals hospitalized in the last 24 hours. The scale consists of 16 items (15 self-report items and one item that results from the sum of the first two). Each item consists of statements with opposite meanings at the end of a straight line of 100 millimeters, divided every five millimeters. A line should be drawn perpendicular to the straight line, in the division that the patient thinks best reflects their situation. It covers three scales: Disturbance (7 items and possible variation from 0 to 700); Effectiveness (5 items, with a maximum of 600, as one of the items, is the result of the sum of two others) and Sleep supplementation (4 items and possible variation from 0 to 400). The values of each scale must be considered separately for the analysis. Thus, the higher the value obtained on the Disturbance and Supplementation scales, the worse the quality of sleep, and, on the Effectiveness scale, the higher score indicates better quality of sleep^8,9^.

The institution’s Research Ethics Committee approved the study under opinion number 1,901,494. The research’s ethical criteria followed Resolution 466/12 of the National Health Council - CNS. All study participants signed the Informed Consent Form (ICF), individually, and in two copies. Due to the use of data contained in the patients’ medical records, we used the Data Use Commitment Term (DUCT).

### Statistical Analysis

The data were inserted into Microsoft Excel® databases. Data processing took place using IBM® SPSS® software, version 23.0. We calculated descriptive statistics such as means, medians, standard deviation, interquartile range, minimums, and maximums, for quantitative variables. The Kolmogorov- Smirnov test verified the normality of the data. The analyzes of the subjective sleep pattern in the last 24 hours and its characteristics were performed using Student’s t-tests, ANOVA One-way, and Pearson’s correlation test. The Kruskal-Wallis test compared the subjective sleep pattern in the last 24 hours and the factors that influence sleep. All analyzes were performed at a 5% significance level.

## RESULTS

The investigation involved the participation of 230 patients of both genders whose ages varied between 18.2 and 88.6 years old, with a mean (± SD) of 46.8 (± 17.2) years old, most of them (57.0%) were female.

Regarding the use of cigarettes and alcohol consumption, 67.8% were non-smokers and 60.9% reported not being alcoholics. The lack of regularity in the practice of physical activity was reported in 83.5% of the patients. The BMI varied between 14.9 to 44.9 kg/ m^2^, with an average of 25.3 (± 5.0) kg/m2. Categorically, patients were divided into low weight (5.2%), normal weight (47.4%), overweight (30.9%), and obesity (16.5%).

The most frequent diagnosis among the clinical causes of hospitalization (61.3%) was systemic lupus erythematosus with 20.6%. Among the surgical causes (38.7%), uterine leukoma stood out with 11.2%. Among the clinical causes of hospitalization (61.3%), the most frequent diagnoses were systemic lupus erythematosus 20.6%, ulcerative colitis 4.3%, idiopathic thrombocytopenic purpura 2.8%, myelodysplastic syndrome 2.8%, neoplasia of lung 2.8% and Crohn’s disease 2.8%. Among the surgical causes (38.7%), the following stood out: uterine leukoma 11.2%, biliary calculus 7.9%, angina pectoris / unstable angina or ICO 7.9%, cholelithiasis 3.4% and umbilical hernia 3.4%. The average hospital stay was 3.0 (± 1.0) days (Table 1).

**Table 1:** Characterization of hospitalized patients (n=230). Teresina, PI, Brazil, 2018.

Characteristics	M	SD	N	%
**Age (years)**	46.8	17.2		
18-28.9			45	19.6
29-39.9			43	18.7
40-49.9			38	16.5
50-59.9			40	17.4
60-69.9			46	20.0
70-79.9			13	5.6
80-89.9			5	2.2
**Gender**				
Male			99	43.0
Female			131	57.0
**Smoking**				
Non-smoker			156	67.8
S.oker			18	7.8
Former smoker			56	24.3
**Alcoholism**				
Yes			42	18.3
No			140	60.9
Ex-alcoholic			48	20.9
**Practice of physical activity**				
Yes			38	16.5
No			192	83.5
**Body mass index (kg/m2)**	25.3	5.0		
Low weight (<18.5)			12	5.2
Normal weigh (18.5-24.9)			109	47.4
Overweight (25-29.9)			71	30.9
Obesity (≥30)			38	16.5
**Cause of hospitalization**				
Clinical			141	61.3
Surgical			89	38.7
**Length of hospital stay (in days)**	3.0	1.0		
48 hours			96	41.7
72 hours			62	27.0
96 hours			45	19.6
120 hours			27	11.7
**Total**			**230**	**100.00**

M: average; SD: standard deviation.

**Figure 1:**
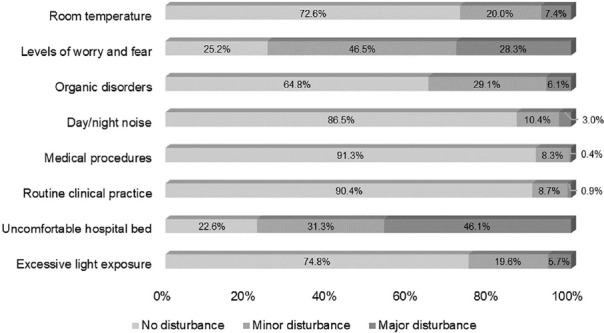
Sleep-disturbing factors assessed by hospitalized patients (n=230). Teresina, PI, Brazil, 2018.

The factors with the highest percentages in the evaluation regarding the highest level of discomfort comprised an uncomfortable bed, followed by feelings of fear and concern, whose values were, respectively, 46.1% and 28.3% (Figure 1).

Regarding the evaluation of the subjective sleep pattern through the (VAS) Scales, an average of 208.7 points was found in the Disturbance scale. The Effectiveness scale had an average of 353.8 points. On the sleep supplementation scale, the mean was 62.9 points (Table 2).

**Table 2:** Descriptive analysis of Visual Analog Sleep (VAS) Scales for hospitalized patients (n=230). Teresina, PI, Brazil, 2018.

Scale/characteristics	Min	Max	M	SD	Md	IIQ
**Disturbance Scale**	**25,0**	**600.0**	**208.7**	**112.3**	**197.5**	**136,3**
Fragmentation characteristics						
Time awake after sleep onset	0.0	100.0	25.1	26.0	15.0	25.0
Sleep depth	0.0	100.0	44.2	39.2	50.0	80.0
Disturbance quality	0.0	100.0	19.7	25.2	10.0	20.0
Awakenings during sleep	0.0	100.0	22.2	26.7	15.0	20.0
Movements during sleep	0.0	100.0	56.6	42.3	55.0	95.0
Latency characteristics						
Sleep latency	0.0	100.0	20.4	27.8	5.0	36.25
Latency quality	0.0	100.0	20.5	29.5	5.0	36.25
**Effectiveness Scale**	**220.0**	**480.0**	**353.8**	**52.2**	**365.0**	**70.0**
Quality characteristics						
Rest after waking up	0.0	100.0	76.1	31.1	90.0	50.0
Subjective sleep quality	0.0	100.0	69.9	32.2	75.0	50.0
Sleep sufficiency evaluation	0.0	100.0	37.3	42.2	10.0	86.25
Duration characteristics						
Total sleeping time	0.0	100.0	72.7	24.5	82.5	20.0
Total sleep period	10.0	165.0	97.8	12.3	100.0	5.0
**Sleep Supplementation Scale**	**0.0**	**400.0**	**62.9**	**67.3**	**40.0**	**75.0**
Sleep time during the day	0.0	100.0	17.8	19.5	15.0	25
Naps in the morning	0.0	100.0	10.3	20.0	5.0	15
Naps in the afternoon	0.0	100.0	18.7	23.5	10.0	25
Time to get up after waking up	0.0	100.0	16.1	27.5	5.0	25

Legend: Min: minimum; Max: maximum; M: mean; SD: standard deviation; Md: median; IIQ: interquartile range.

When analyzing the relationships between the subjective sleep pattern in the last 24 hours and the clinical characteristics of hospitalized patients, a statistically significant difference was identified between the mean of the scores on the Disorder scale and the practice of physical activity (p = 0.024). There was also a statistically significant relation between BMI and sleep effectiveness (p = 0.028)(Table 3).

**Table 3:** Relationships between the subjective pattern of sleep in the last 24 hours and the clinical characteristics of hospitalized patients (n=230). Teresina, PI, Brazil, 2018.

Characteristic	Disturbance		Effectiveness		Sleep supplementation	
	M (±DP)	*P*	M (±DP)	*p*	M (±DP)	*p*
**Age(years)**	-	0.738^a^	-	0.918^a^	-	0.702^a^
18-28.9	214.2 (120.5)		351.4 (53.7)		71.7 (72.2)	
29-39.9	210.9 (128.3)		348.5 (58.6)		64.9 (61.7)	
40-49.9	216.7 (107.5)		356.0 (54.9)		63.3 (59.1)	
50-59.9	196.0 (96.2)		358.4 (49.3)		52.4 (64.5)	
60-69.9	219.9 (114.5)		351.1 (50.0)		66.3 (83.5)	
70-79.9	161.9 (101.4)		366.1 (44.4)		61.1 (44.3)	
80-89.9	199.0 (54.1)		366.0 (29.0)		21.0 (14.7)	
**Gender**		0.434^t^		0.149^t^		0.209^t^
Female	213.7 (114.6)		349.6 (53.9)		67.6 (73.3)	
Male	202.0 (109.4)		359.6 (49.3)		56.7 (58.2)	
**Smoking**		0.513^t^		0.051^t^		0.134^t^
Current or previous	202.4 (89.2)		363.7 (48.5)		53.8 (58.3)	
No	211.7 (121.9)		349.3 (53.3)		67.2 (70.9)	
**Alcoholism**		0.477^t^		0.858^t^		0.535^t^
Current or previous	215.3 (106.9)		353.2 (53.4)		59.4 (63.6)	
No	204.5 (115.8)		354.4 (51.5)		65.1 (69.7)	
**Physical activity**		0.024^t^		0.278^t^		0.581^t^
Yes	252.2 (129.0)		345.5 (52.4)		57.4 (51.1)	
No	200.1 (106.9)		355.6 (52.1)		63.9 (70.1)	
**BMI**	-	0.662^a^	-	0.028^a^	-	0.410^a^
Low weight (<18.5)	176.7 (100.1)		376.7 (35.4)		59.6 (45.1)	
Normal weight (185-24.9)	213.1 (118.5)		357.7 (52.0)		64.7 (79.0)	
Overweight (25-29.9)	202.5 (106.0)		348.7 (54.9)		53.6 (47.9)	
Obesity (≥30)	217.6 (110.7)		345.5 (50.3)		76.0 (67.1)	
**Cause of hospitalization**		0.346^t^		0.660^t^		0.782^t^
Clinical	214.3 (114.5)		355.1 (54.5)		61.9 (72.6)	
Surgical	199.9 (108.7)		352.0 (48.4)		64.4 (58.2)	
**Length of hospital stay**		0.159^a^		0.144^a^		0.168^a^
48 hours	206.9 (107.6)		359.7 (52.3)		54.1 (58.4)	
72 hours	196.7 (100.5)		350.1 (49.7)		67.7 (58.7)	
96 hours	240.7 (135.7)		340.8 (51.6)		79.6 (86.1)	
120 hours or more	189.4 (106.6)		364.1 (55.7)		55.6 (77.1)	

BMI: body mass index; M: mean; SD standard deviation; *p*: significance of the test; t: Student's t test; a: ANOVA One-way test.

**Figure 2:**
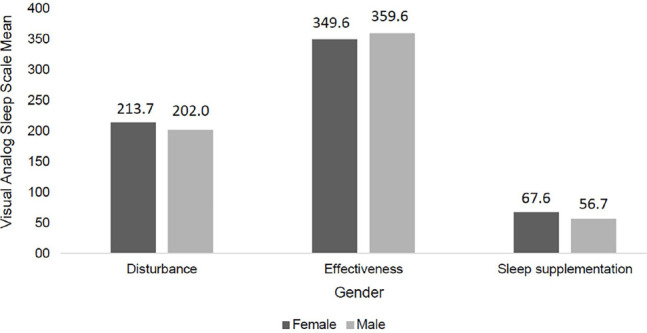
Relationship between sex and the subjective sleep pattern in the scales of sleep disturbance, effectiveness and supplementation (n=230). Teresina, PI, Brazil, 2018.

**Figure 3:**
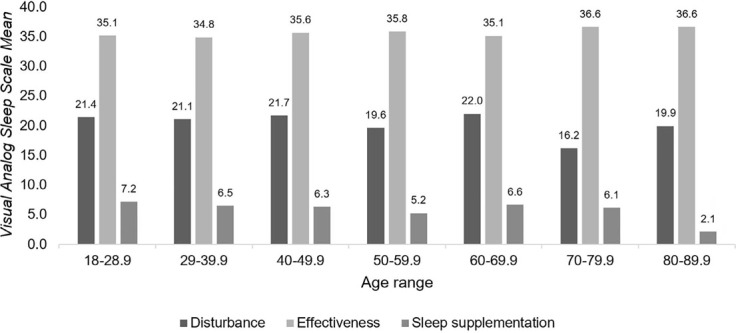
Relationship between age groups and the subjective sleep pattern in the scales of sleep disturbance, effectiveness and supplementation (n=230). Teresina, PI, Brazil, 2018.

Table 3 also shows the analyzes differentiated by sex and age group, with no statistically significant differences being found between these variables and the subjective sleep pattern in the sleep disorder, effectiveness and supplementation scales. For better observation, the analyzes were evidenced through figures 2 and 3.

We observed a statistically significant differences between the VAS scores for Disturbance and Effectiveness and factors influencing sleep. Both interfered with excessive lighting (p = 0.001 and p = 0.023), uncomfortable bed (p <0.001 and p <0.001) and organic disorders (p <0.001 and p = 0.001) (table 4).

**Table 4:** Relationships between the subjective sleep pattern in the last 24 hours and the sleep influencing factors that caused discomfort in the evaluation of hospitalized patients (n=230). Teresina, PI, Brazil, 2018.

Environment factor*	Disturbance	Effectiveness*	Sleep supplementation*
Excessive lighting	0.001	0.023	0.617
Uncomfortable bed	<0.001	<0.001	0.066
Infirmary routine	0.080	0.866	0.854
Care Received	0.182	0.233	0.508
Noise in the infirmary	0.674	0.383	0.855
Organic disorders	<0.001	0.001	0.324
Fear and worry	0.069	0.757	0.975
Room temperature	0.588	0.881	0.585

*: Kruskal-Wallis test.

## DISCUSSION

During the hospitalization, there was a certain degree of perception in which the main sleep time was impaired due to sleep fragmentation and latency (disorder) and that the level of sleep effectiveness the previous night was not high enough, requiring sleep supplementation. during the day. Factors that interfered with sleep during hospitalization were: the practice of regular physical activity, higher body mass index, and factors influencing sleep.

Female individuals (57%) and with a mean age of 46.8 years old prevailed in the study. The analysis in the literature proves that, conversely, the females have been the least frequent in research that seeks to identify the subjective sleep pattern in hospitalized patients^8,9,10^,^11,12^. The average age found in patients differed from what is commonly found in studies on sleep in a hospital environment, which, in its majority, is over 60 years old^13,14^.

The lack of regularity in the practice of physical activity was high (83.5%) and the average BMI was 25.3 kg/m^2^, with an important percentage of overweight and obesity, being almost half of the sample (47.4%). Studies have identified an association between sleep disorders and the presence of overweight or obesity^15,16,17^. Sleep disorders and shorter sleep duration are related to weight gain; and the decrease in sleep quality seems to be associated with greater food intake^18^. On the other hand, the improvement in sleep quality seems to be mediated by the practice of regular physical activity^19^.

Uncomfortable bed, fear, and worry were the factors that most bothered patients among the reported factors that interfered with night sleep. Negative evaluations regarding bed discomfort were just under 80%, ranging from little (31.3%) to very uncomfortable (46.1%).

Hospitalization is a difficult time for the patient because, in addition to the organic problems that led to hospitalization, there is psychological stress generated in such situations and the hospital environment can be harmful to the induction and maintenance of a normal sleep-wake cycle. The uncomfortable bed was reported as a reason for poor sleep by 18.0% of patients in a study in Canada^13^. However, in a study conducted in Brazil, the authors observed a few complaints regarding the discomfort caused by the hospital bed^1^.

In another study, emotional factors such as concern about the disease and being in the hospital were the most important aspects that negatively interfered with sleep during hospitalization^4^. Thus, the authors highlighted the hospital environment as stressful, where there is an increase in anxiety and pain perception, decreasing sleep, and prolonging recovery.

The sleep analysis in the hospital environment using the VAS Scales showed an average of the scores on the Disturbance scale of 208.7 points, corresponding to about 29.8% of the maximum perception of the degree in which the main time of the sleep was impaired due to fragmentation and sleep latency. The fragmentation characteristics identified had important degrees of movement in the bed throughout the night and light sleep, respectively in the sample of hospitalized patients. Latency quality data expressed the presence of some level of difficulty in falling a sleep.

Studies have been showing the impacts of findings such as those mentioned above on the health of hospitalized patients. Sleep deprivation in hospitalized patients has serious consequences and can contribute to the deterioration of their condition. Changes in the sleep-wake cycle cause not only poor sleep quality and a feeling of tiredness the next day but also changes in the body’s circadian biorhythms, as it disrupts the activity of normal physiological processes, which can hinder the patient’s recovery^20^.

Sleep fragmentation, found in the present research, in addition to other negative factors, causes immunological dysfunction, predisposing less resistance to infections and changes in the blood coagulation cascade, and delayed wound healing^21^. Changes in the sleep-wake pattern cause changes in patients’ homeostasis and well-being, decreasing the potential for improvement and increasing the number of days of hospitalization^22^.

The Effectiveness scale had an average value of 353.8 points, and the measure corresponds to approximately 59% of the maximum perception of the degree of effectiveness of the main sleep time. Rest on waking (perception of how rested the person feels upon waking up) was the characteristic that most contributed to the quality of sleep; while the total sleep period (perception of the total time spent in bed trying to sleep) was the factor that most interfered with the sleep duration characteristic.

Regarding the Sleep Supplementation scale, the average score was 62.9 points, corresponding to 15.7% of the maximum perception of the degree in which additional periods of sleep increased the main sleep time. The main characteristics that influenced this evaluation included naps in the afternoon and total sleep time during the day.

In a study with elderly people admitted to a university hospital in the state of São Paulo, Brazil, the values obtained with the VAS Scales revealed scores very similar than in this research, in which the averages referring to the Disturbance, Effectiveness and Supplementation scales were, 294.8; 374.0 and 98.1 points respectively^8^.

The analysis of 100 adults admitted to a general hospital in Canada obtained worse values with the VAS Scales in this study, showing by the higher Disturbance (mean: 350) and Supplementation (mean: 100) measures and slightly lower Effectiveness (average: 300)23. Another study also carried out in Canada showed poor sleep quality, measured by the Disturbance (average: 371), Effectiveness (average: 190), and Supplementation (average: 115) scales13.

In this study, the percentage corresponding to less effectiveness was high (41.0%), causing the need to supplement sleep during the day (15.7%). From these supplementation data, there is some loss in the effectiveness of sleep during the previous night during the hospitalization period. Although the average value of the Supplementation Scale was low, the need to complement sleep was evident with naps during the day.

According to the data of this research, patients who regularly practiced some physical activity before hospitalization had a worse quality of sleep in the hospital environment in the last 24 hours, on the Disturbance scale, when compared to those who did not practice it.

Physical activity positively interferes with sleep quality, especially in circadian rhythm disorders, so that regular physical activity seems to increase the depth and duration of sleep^24^. A longitudinal study carried out in China with a sample of 1224 adults, found that the practice of physical activity was characterized as a preventive factor against sleep disorders^19^.

The inverse correlation between BMI and the Effectiveness scale score showed that patients with higher body mass values (overweight and obesity) had worse sleep effectiveness. These findings corroborate with a Chinese study with the multivariate logistic regression model, that female gender, overweight, obesity, and sleep duration were independent predictors of worse sleep quality^25^.

The factors in this study, which significantly interfered with the Disturbance and Effectiveness scales were: uncomfortable bed, organic disturbances, and excessive lighting. The structure of the bed and pillow (72.3%) and the lighting of the room at night (72%) ^26^; and organic disorders (36.8%) are the frequently self-reported causes of sleep interruption in the hospital^1^.

It is noteworthy that thelight is the main environmental signal for the timing of the circadian cycle^12^ and plays a vital role in the timing of the circadian cycle^7^. Environmental variables such as the exposure to light in the morning (for example, natural lighting from open windows) work as a “time giver” (*zeitgeber*)^27^ for the suprachiasmatic nucleus, allocating the sleep phase in the night shift accordingly. In contrast, excessive nighttime artificial lighting can awaken the individual, as it increases alert levels and interferes with the release of hormone regulating the sleep-wake cycle.

The limitations of this study consist in the fact that evaluations by measuring instruments depend on the elements of memory and authenticity of the answers provided by the patients. Small divergences from what occurs can influence the interpretation of the data mathematically. Because it is a nonexperimental study and also a psychological object evaluation, there is less control of variables. Another limitation of the study was the lack of verification of the time of sleep onset, awakening, duration of night sleep and duration of naps for a better characterization of the study participants, however the best instrument for applicability in the hospital environment was sought.The results of this research provoke reflections on the importance of planning strategies to promote sleep quality, showing that the interaction between patient, multidisciplinary team, and hospital management is essential in the early identification of sleep disorders, as well as in the reduction of factors that interfere with sleep during hospitalization.

Evidence shows thatfavorable environment that facilitates the onset of sleep reduces the number of interruptions and enables to maintain the sleep-wake cycle is essential to obtain quality sleep in a hospital environment. Rotines should be planned not disturbing patients’ sleep or interrupt it frequently, as well as adjustments in patients’ rooms to generate more comfort require multidisciplinary cooperation and hospital management participation.

Therefore, we concluded that there was a certain degree of sleep disturbance during the hospitalization period and the percentage of the less sleep effectiveness was relatively high, causing the need to supplement sleep during the day. Also, some factors in particular negatively influenced the quality of sleep in the hospital.

## References

[1] Costa SV, Ceolim MF (2013). Fatores que interferem na qualidade do sono de pacientes internados. Rev Esc Enferm USP.

[2] Dogan O, Ertekin S, Dogan S (2005). Sleep quality in hospitalized patients. J Clin Nurs.

[3] Reid E (2001). Factores affecting how patients sleep in the hospital environment. Br J Nurs.

[4] Silva LE, Oliveira ML, Inaba WK (2011). Fatores que interferem na qualidade do sono de pacientes internados. Rev Eletr Enf.

[5] Besedovsky L, Lange T, Bom J (2012). Sleep and immune function. Pflugers Arch.

[6] Lashley FR, Frank-Stromborg M, Olsen SJ (2004). Instruments for clinical health-care research.

[7] Beltrami, FG, Nguyen X, Pichereau C, Maury E, Fleury B, Fagondes S (2015). Sleep in the intensive care unit. J. Bras. Pneumol.

[8] Monteiro NT, Ceolim MF (2014). Qualidade do sono de idosos no domicílio e na Hospitalização. Texto Contexto Enferm.

[9] Bergamasco EC, Cruz DALM (2007). Adaptação da Visual Analog Sleep Scales para a língua portuguesa. Rev. Latino Am. Enferm.

[10] World Health Organization (2000). Obesity: preventing and managing the global epidemic. Report of a World Health Organization Consultation. Geneva: World Health Organization. WHO Obesity Technical Report Series.

[11] Arora VM (2011). Objective sleep duration and quality in hospitalized older adults: associations with blood pressure and mood. J Am Geriatr Soc.

[12] Bano M (2014). The influence of environmental factors on sleep quality in hospitalized medical patients. Frontiers Neurol.

[13] Dobing S (2016). Frolova N, McAlister F, Ringrose J. Sleep Quality and Factors Influencing Self-Reported Sleep Duration and Quality in the General Internal Medicine Inpatient. PLoS One.

[14] Adachi M, Staisiunas PG, Knutson KL, Beveridge C, Meltzer DO, Arora VM (2013). Perceived control and sleep in hospitalized older adults: a sound hypothesis?. Hosp Med April.

[15] Beccuti G, Pannain S (2011). Sleep and obesity. Curr Opin Clin Nutr Metab. Care.

[16] Kim SK (2012). Smoking induces oropharyngeal narrowing and increases the severity of obstructive sleep apnea syndrome. J Clin Sleep Med.

[17] Peters EN, Fucitoa LM, Novosadb C, Toll BA, O’Malleya SS (2011). Effect of night smoking, sleep disturbance, and their co-occurrence on smoking outcomes. Psychol. Addict Behav.

[18] Galli G (2013). Inverse relationship of food and alcohol intake to sleep measures in obesity. Nutr Diabetes.

[19] Zuo H, Shi Z, Yuan B, Dai Y, Hu G, Wu G, Hussain A (2012). Interaction between physical activity and sleep duration in relation to insulin resistance among non-diabetic Chinese adults. BMC Public Health.

[20] Nerbass FB, Peruchi BB, Martins JA, Andrade FM, Dias CM (2015). Associação Brasileira de Fisioterapia Cardiorespiratória e Fisioterapia em Terapia Intensiva.

[21] Pulak LM, Jensen L (2014). Sleep in the Intensive Care Unit: A Review. J Intensive Care Med.

[22] Pinto JP (2014). Estudo da qualidade do sono dos pacientes internados no serviço de cirurgia e de medicina interna do Centro Hospitalar Cova da Beira.

[23] Frighetto L, Marra C, Bandali S, Wilbur K, Naumann T, Jewesson P (2004). An assessment of quality of sleep and the use of drugs with sedating properties in hospitalized adult patients. Health Qual. Life Outcomes.

[24] Ohayon MM, Carskadon MA, Guilleminaut C, Vitiello MV (2004). Meta-analysis of quantitative sleep parameters from child-hood to old age in healthy individuals: developing normative sleep values across the human lifespan. Sleep.

[25] Hung HC, Yang YC, Ou HY, Wu JS, Lu FH, Chang CJ (2013). The association between self-reported sleep quality and overweight in a Chinese population. Obesity.

[26] Yilmaz M, Sayin Y, Gurler H (2012). Sleep quality of hospitalized patients in surgical units. Nurs Forum.

[27] Lima LE, Vargas NN (2014). O Relógio Biológico e os ritmos circadianos de mamíferos: uma contextualização histórica. Rev Biol.

